# The temperature sensitivity of soil organic carbon decomposition is greater in subsoil than in topsoil during laboratory incubation

**DOI:** 10.1038/s41598-017-05293-1

**Published:** 2017-07-12

**Authors:** Dong Yan, Jinquan Li, Junmin Pei, Jun Cui, Ming Nie, Changming Fang

**Affiliations:** Coastal Ecosystems Research Station of the Yangtze River Estuary, Ministry of Education Key Laboratory for Biodiversity Science and Ecological Engineering, The Institute of Biodiversity Science, Fudan University, Shanghai, 200433 PR China

## Abstract

The turnover of soil organic carbon (SOC) in cropland plays an important role in terrestrial carbon cycling, but little is known about the temperature sensitivity (*Q*_10_) of SOC decomposition below the topsoil layer of arable soil. Here, samples of topsoil (0–20 cm) and subsoil (20–40 cm) layers were obtained from paddy fields and upland croplands in two regions of China. Using a sequential temperature changing method, soil respiration rates were calculated at different temperatures (8 °C to 28 °C) and fitted to an exponential equation to estimate *Q*_10_ values. The average SOC decomposition rate was 59% to 282% higher in the topsoil than in the subsoil layer because of higher labile carbon levels in the topsoil. However, *Q*_10_ values in the topsoil layer (5.29 ± 1.47) were significantly lower than those in the subsoil layer (7.52 ± 1.84). The pattern of *Q*_10_ values between the topsoil and subsoil was significantly negative to labile carbon content, which is consistent with the carbon quality-temperature hypothesis. These results suggest that the high temperature sensitivity of SOC decomposition in the subsoil layer needs to be considered in soil C models to better predict the responses of agricultural SOC pools to global warming.

## Introduction

Soils contain approximately 1,500 Pg organic carbon (C) in the global upper 100 cm, which is about three times the amount stored in terrestrial vegetation (550 Pg) and twice that stored in the atmosphere (750 Pg)^1, 2^. The soil organic carbon (SOC) pool plays important roles in the cycling and balance of global C^3^. The global storage of SOC in cropland is about 128–165 Pg C^4^, which is approximately 8% to 10% of the terrestrial SOC pool^5, 6^. In addition to being an important part of global SOC storage, SOC in cropland is the most active SOC pool among terrestrial ecosystems^7^.

Global warming has already had observable effects on the environment^8^. Although warming is expected to accelerate SOC decomposition, the responses of SOC to warming still exhibit many uncertainties^3^. One of the key factors leading to these uncertainties is the temperature sensitivity (*Q*_10_) of SOC decomposition^3^. SOC storage in cropland is not only influenced by climate change, but is also regulated by human activities over a short period of time. Therefore, understanding the *Q*_10_ of SOC decomposition in cropland is important for understanding global C cycling. Unlike natural soils, cropland soils have relatively low organic C concentrations, and thus a higher C sequestration potential^4^. Sustainable land use and management practices in agroecosystems could have the potential to sequester approximately 55–78 Pg SOC^9^.

Many studies have focused on soil respiration in croplands (e.g. Lohila *et al*.^10^, Fiener *et al*.^11^, and Campos^12^). Field-measured soil respiration is the CO_2_ flux emitted from the soil surface, which commonly includes both autotrophic and heterotrophic respiration^13^, with autotrophic respiration occupying 30% to 70% of the total^14, 15^. Therefore, field measurements can not clearly distinguish between SOC decomposition from topsoil (TL) as opposed to subsoil layers (SL). In laboratory soil incubation experiments, live roots are commonly removed from soil samples^16, 17^. The measured CO_2_ flux in this case is attributed to heterotrophic respiration, i.e., SOC decomposition^18^, reflecting the SOC decomposition potential under the specified conditions. For example, Arevalo *et al*.^19^ reported that the amount of mineralized C from root-free soil over a 370-d incubation period ranged between 2% and 9% of the initial total SOC in four land use system soils. However, the cropland soil samples used in most studies have been collected from the TL (e.g. Iqbal *et al*.^16^, Thiessen *et al*.^17^, and Guntinas *et al*.^20^). Little is known about organic C decomposition in the SL in cropland^21^.

Because of differences in the intensity of human disturbances, cropland soils have distinct soil layers: TL and SL. The TL is often disturbed by ploughing and other agricultural activities^22^ and is characterized by a porous soil structure with high permeability^23^, rapid changes in moisture and temperature^24^ and abundant nutrients and organic materials from crop root turnover and exudation^25^. TL compaction by mechanical farm operations causes the subsoil to have a dense and hard pan (commonly referred to as plough sole) of high bulk density^26^, restricting the exchange of gas, water, nutrients and other substances between soil layers^27, 28^. Moreover, variations in temperature and water conditions are smaller in the SL than in the TL^29^. Thus, SOC pools, decomposition, and dynamics may differ between TL and SL^30, 31^.

SOC stored in the SL as well as in the TL plays an important role in SOC storage within the entire soil profile^32, 33^. For example, Xie *et al*.^34^ reported that SOC in the surface and subsurface layers constituted about 30% and 70%, respectively, of the total SOC pool up to 1 m depth in Chinese croplands. Jenkinson and Coleman^35^ found that treating the entire soil profile (1 m depth) as a homogenous unit, instead of dividing it into different soil horizons, overestimates the impact of global warming on C decomposition. It is therefore essential to consider the differences in SOC decomposition in different soil layers in predictive models.

In this study, we aimed to estimate differences in SOC decomposition and its temperature sensitivity between topsoil and subsoil layers in paddy-upland rotation and upland farming systems in China. We hypothesized that SOC quality in the TL would be higher than in the SL. Because low-quality soil C is more sensitive to temperature change than high-quality soil C^3^, we also expected that SOC in the SL would be more sensitive to temperature increases than SOC in the TL in both paddy-upland rotation and upland farming systems.

## Results

### Soil properties and soil respiration

SOC content was significantly higher in the TL than in the SL across sites (Table 1; *P* < 0.05). On average, the SOC in the TL was 14.2 g kg^−1^ for paddy field and 13.0 g kg^−1^ for upland soil, while the values in the SL were 12.1 g kg^−1^ and 9.9 g kg^−1^ for paddy field and upland soil, respectively. Furthermore, permanganate oxidizable C (POXC) as an index of soil labile C was significantly higher in the TL than in the SL for both paddy field and upland soil (Table 1; *P* < 0.05), suggesting that SOC had a higher quality in the TL than in the SL. The POXC in the TL was 3.2 g kg^−1^ for paddy field soil and 1.5 g kg^−1^ for upland soil, which was about 154% and 122% higher than in the paddy field and upland soil in the SL, respectively. Soils in the Meishan (MS) area had significantly higher N content than those in the Huaping (HP) area (Table 1), possibly because of different agricultural practices in the two areas. The soil physicochemical properties of each soil sample are listed in the Supplementary Tables.

**Table 1:** Soil physicochemical properties of the topsoil layer (TL) and subsoil layer (SL).

Area	Land use	Soil layer	pH (H_2_O)	WHC %	SOC g kg^−1^	POXC g kg^−1^	TN g kg^−1^	C/N
HP	Paddy field	TL	8.1(0.3)	27.6 (1.6)	14.1 (2.0)	2.5(0.4)	1.1 (0.2)	16.2 (0.8)
		SL	8.3(0.2)	28.5 (2.3)	12.4 (1.6)	1.1(0.3)	0.9 (0.2)	16.3 (1.4)
	Upland	TL	8.5 (0.2)	26.4 (2.2)	8.0 (3.6)	0.7(0.2)	0.7 (0.3)	13.1 (0.3)
		SL	8.6 (0.2)	29.0 (2.0)	4.5 (2.4)	0.3(0.1)	0.4 (0.2)	14.0 (0.9)
MS	Paddy field	TL	5.9 (0.2)	31.4 (3.3)	14.3 (3.6)	3.8(0.5)	1.2 (0.3)	12.3 (0.5)
		SL	6.1 (0.2)	32.5 (3.3)	11.9 (2.9)	1.4(0.3)	1.0 (0.2)	11.9 (0.6)
	Upland	TL	6.3 (0.3)	34.3 (3.4)	18.0 (7.1)	2.3(0.8)	1.4 (0.6)	13.4 (0.6)
		SL	6.4 (0.3)	33.6 (3.7)	15.3 (7.0)	1.1(0.3)	1.3 (0.6)	12.6 (0.4)

Values are means of six sampling sites in each area (Huaping, HP and Meishan, MS). Numbers in parentheses are standard deviations (n = 6).

### Soil respiration and its temperature sensitivity (*Q*_10_)

**Figure 1:**
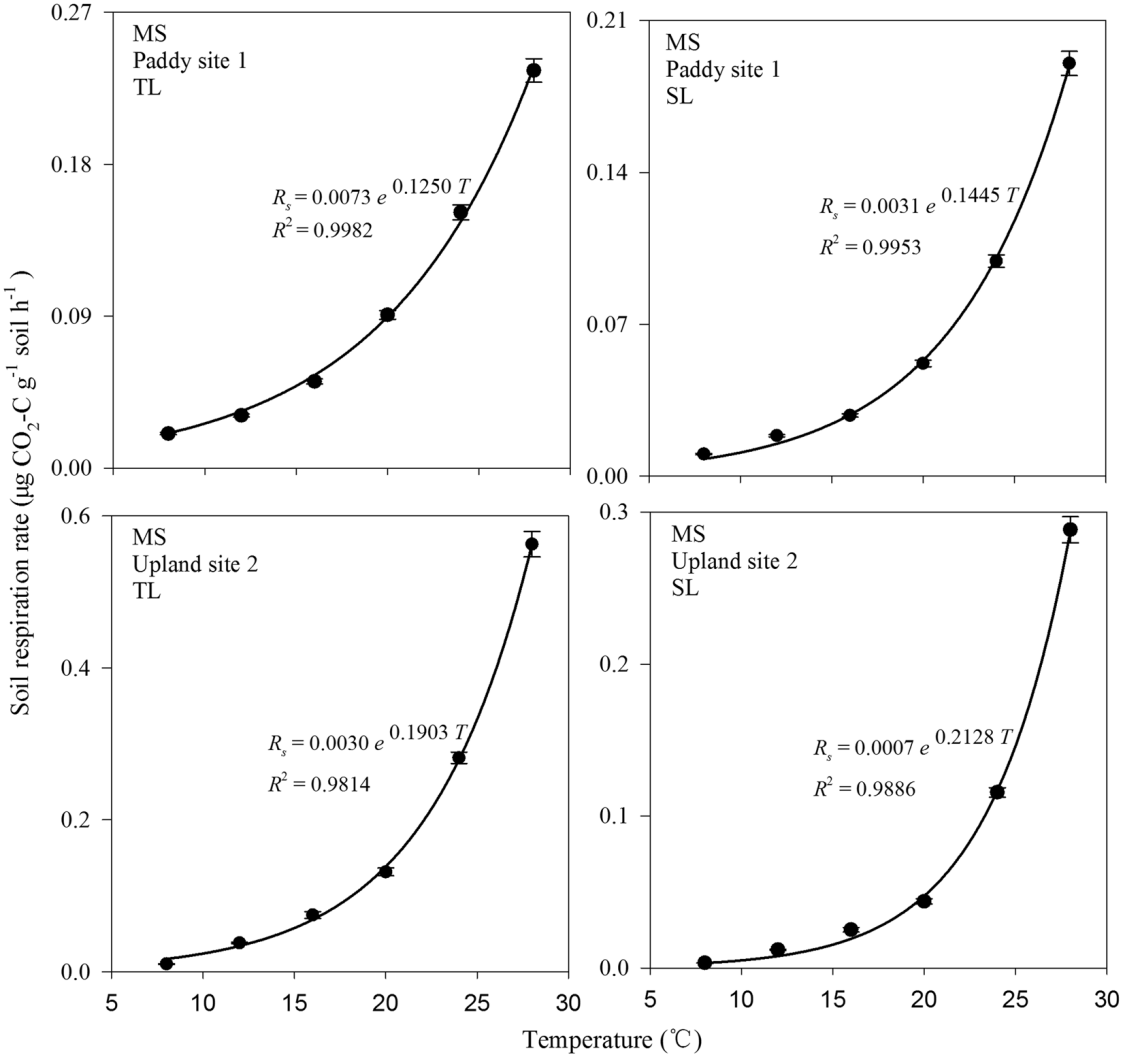
Variation of respiration rate with incubation temperature. Error bars indicate standard deviation (n = 4). TL, SL, and MS represent topsoil layer, subsoil layer, and Meishan, respectively.

**Figure 2:**
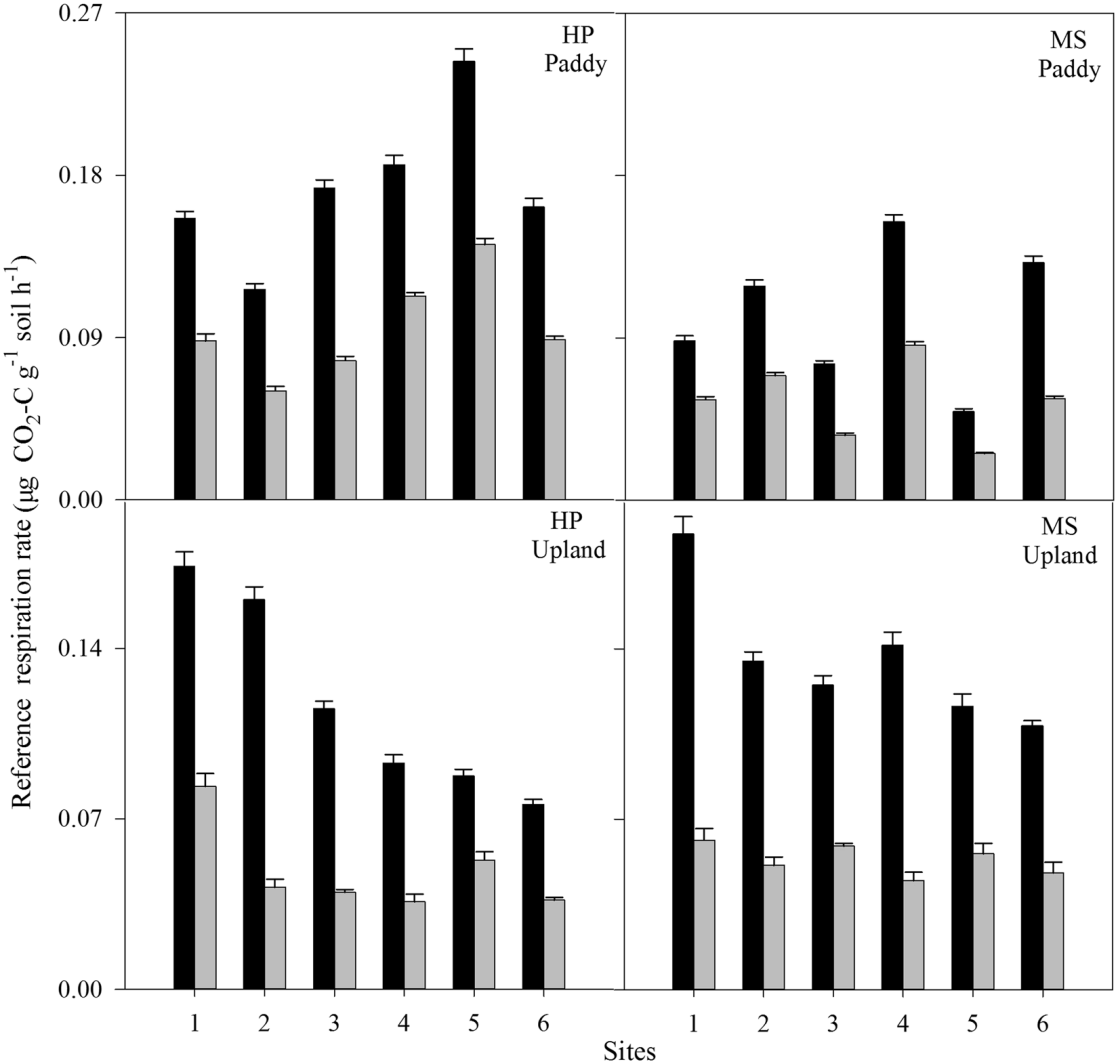
SOC decomposition rate (at 20 °C) in topsoil layer (TL, black bars) and subsoil layer (SL, gray bars). Error bars indicate standard deviation (n = 4). Means are the average decomposition rates for six sites. HP and MS represent Huaping and Meishan, respectively.

The relationship between soil respiration and temperature fitted well to an exponential model (Eq. (2)), with *R* ^2^ varying from 0.95 to 0.99 for all the samples (Fig. 1). For paddy field soil, the SOC decomposition rate at 20 °C (*R*_20_) in the TL (0.0492~0.2431 μg CO_2_-C g^−1^ soil h^−1^) was about 84% higher than in the SL (0.0259~0.1415 μg CO_2_-C g^−1^ soil h^−1^); for upland soil, the value in the TL (0.0759~0.1873 μg CO_2_-C g^−1^ soil h^−1^) was about 149% higher than in the SL (0.0360~0.0833 μg CO_2_-C g^−1^ soil h^−1^) (Fig. 2). Two-factor analysis of variance showed that the SOC decomposition rate at 20 °C in the TL was significantly higher than in the SL (*P* < 0.01).

**Figure 3:**
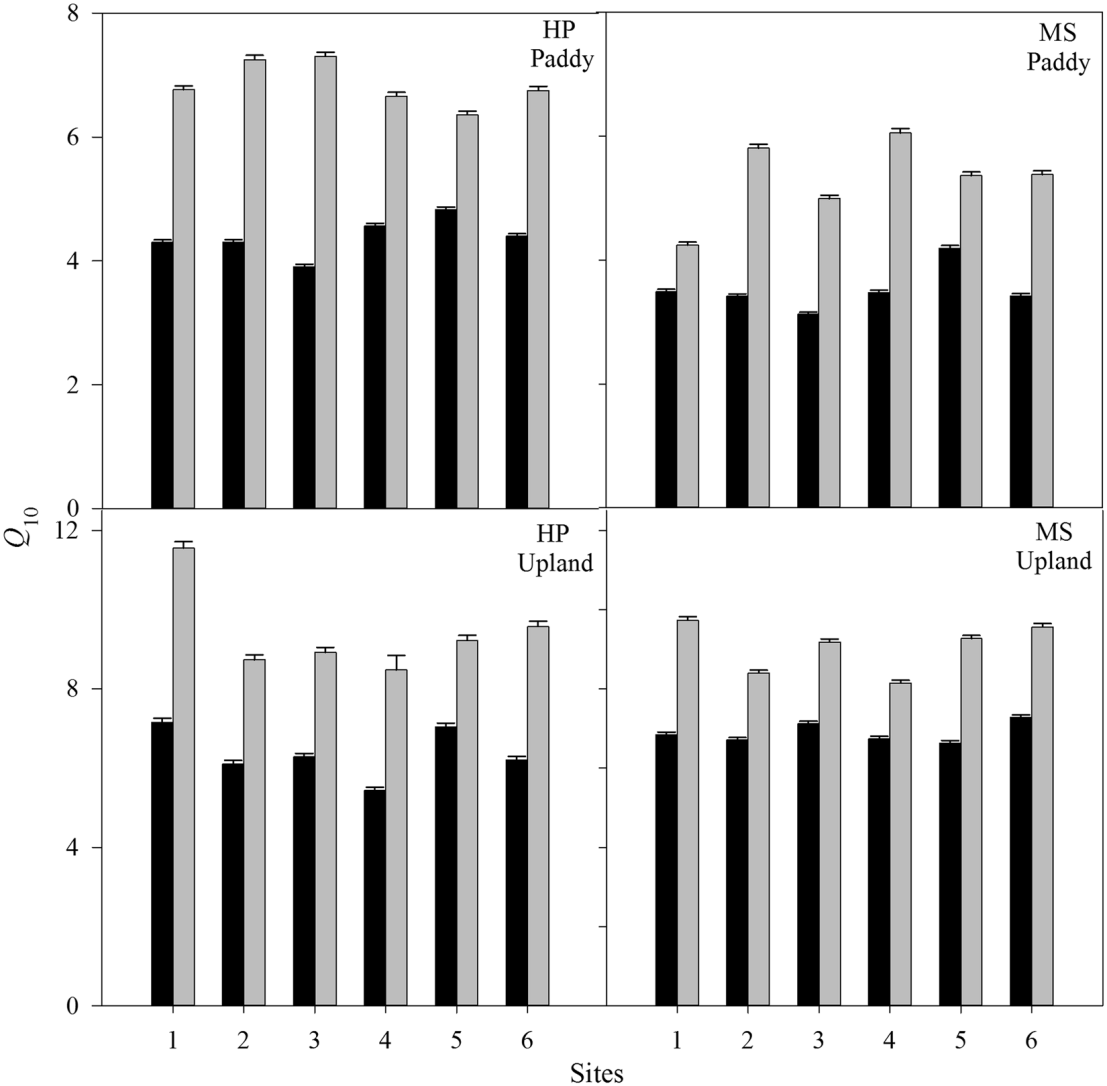
The *Q*_10_ value for topsoil layer (TL, black bars) and subsoil layer (SL, gray bars) of paddy field and upland soils. Error bars indicate standard deviation (n = 4). Means are average *Q*_10_ values for six sites. HP and MS represent Huaping and Meishan, respectively.

For each sampling site, *Q*_10_ values for SOC decomposition in the TL were significantly lower than those in the SL regardless of different land use types (Fig. 3). On average, *Q*_10_ values in the TL were 55 ± 20% and 40 ± 13% lower than those in the SL for paddy field and upland soil, respectively. In the paddy field, the mean *Q*_10_ values in the TL were 4.38 ± 0.31 and 3.52 ± 0.36 for HP and MS, respectively, and those in the SL were 6.85 ± 0.36 and 5.31 ± 0.64, respectively. In the upland soils, the average *Q*_10_ values in the TL were 6.37 ± 0.64 and 6.88 ± 0.26, respectively, and in the SL they were 9.41 ± 1.11 and 9.05 ± 0.64, respectively. SOC decomposition in the paddy field soil was significantly less sensitive to temperature changes than in the upland soil. The *Q*_10_ values averaged over sites and soil layers were 5.14 ± 1.33 and 8.05 ± 1.54 for the paddy field and upland soils, respectively.

### Correlations

In order to eliminate the influences of soil type (paddy field soil and upland soil) and sampling area (HP and MS), we assessed the correlations between *R*_20_ and POXC, and between *Q*_10_ and POXC, for each soil type in each location. The results showed that POXC was positively correlated with *R*_20_ for each soil type at each location (Table 2). However, the correlations between *Q*_10_ and POXC were negative and significant for the two soil types at both HP and MS (Table 3).

**Table 2:** Pearson correlation coefficients (*r*) between *R*_20_ values and POXC.

	HP		MS	
	*r*	*P*	*r*	*P*
Paddy field	0.685	**0**.**014**	0.679	**0**.**015**
Upland	0.679	**0**.**015**	0.792	**0**.**002**
All	0.726	**0**.**000**	0.567	**0**.**004**

HP and MS represent Huaping and Meishan, respectively (n = 12).

**Table 3:** Pearson correlation coefficients (*r*) between *Q*_10_ values and POXC.

	HP		MS	
	*r*	*P*	*r*	*P*
Paddy field	−0.971	**0**.**000**	−0.941	**0**.**000**
Upland	−0.857	**0**.**000**	−0.784	**0**.**003**
All	−0.865	**0**.**000**	−0.727	**0**.**000**

HP and MS represent Huaping and Meishan, respectively (n = 12).

## Discussion

On average, *R*_20_ in the TL was 0.1728 ± 0.0416 (mean ± SD) μg CO_2_-C g^−1^ soil h^−1^ for paddy field soil and 0.1030 ± 0.0389 μg CO_2_-C g^−1^ soil h^−1^ for upland soil; this was significantly higher than in the SL where the mean values were 0.0484 ± 0.0182 μg CO_2_-C g^−1^ soil h^−1^ and 0.0534 ± 0.0065 μg CO_2_-C g^−1^ soil h^−1^ for paddy field and upland soil, respectively. Firstly, fresh organic matter derived from residues, root secretions, and/or organic fertilizer is often concentrated in the surface layer^36^, leading to a higher POXC in the TL than in the SL (Table 1). Secondly, the differences bwtween *R*_20_ in the TL and the SL may be caused by differences in soil microbes. Babujia *et al*.^37^ found significantly higher microbial biomass in the TL than in the SL of soybean-wheat rotation soils. Though experimental conditions, especially oxygen conditions, were the same between the TL and the SL in our study, there might be more anaerobic and inactive microbes in the SL relative to the TL^38^. A study by van Leeuwen *et al*.^39^ also showed that microbial biomass and activity decreased with soil depth in cropland.

In addition, *R*_20_ was significantly higher in paddy field soils than in upland soils, except in the TL of the MS sites where *R*_20_ was not significantly different between paddy field and upland soils. This pattern of *R*_20_ may be due to higher SOC content in paddy fields relative to uplands (Table 1) and it is consistent with previous studies of cropland soils^40^. The SOC content in the upland soils of the MS sites was the highest among all the sites.

*Q*_10_ is a critical factor in predicting future changes in soil C pools^41^. Although field studies more closely replicate natural conditions than do laboratory studies, *Q*_10_ values for soil respiration from field studies are usually confounded by variation in root respiration and other environmental factors (e.g., substrate and water conditions). Although laboratory incubation is commonly criticized for being unnatural^42^, it is an effective complementary way to study the *Q*_10_ of SOC decomposition^43^. In this study, the *Q*_10_ values of cropland soil at depths of 0–20 cm and 20–40 cm ranged from 3.1 to 11.6, with an average of 6.5, which was higher than the commonly reported *Q*_10_ values (1.73~4.67)^19, 44, 45^. These differences in *Q*_10_ values may be the result of differences in the incubation methods used in this study and in others. Zhu and Cheng found that *Q*_10_ values were higher under constant temperatures than under diurnally-varying temperatures in farm and grassland soils through a 122-day incubation^46^. In most reported studies, soil samples were incubated at two or three constant temperatures to estimate the *Q*_10_ value (e.g., Arevalo *et al*.^19^, Conant *et al*.^45^, and Haddix *et al*.^47^). However, this approach has shortcomings. Firstly, it may underestimate the *Q*_10_ value^48^, because the depletion rates of C substrate incubated at different temperatures are different, with samples incubated at relatively high temperatures using up the more labile fractions of SOC sooner than those incubated at lower temperatures^49^. Secondly, microbies may adapt to different constant temperatures during the long-term incubation, which may lead to contradictory measurements of *Q*_10_ values^3^. The uncertainties in the estimated temperature sensitivity of SOC decomposition caused by the methodology used have not yet been examined, and the underlying mechanisms are not yet known. Further studies are warranted to reliably predict the feedback between soil C storage and global climate change.

The decomposition of SOC in upland soil had higher *Q*_10_ values than the decomposition in paddy field soil, and this pattern was consistent across sampling sites in both the HP and MS regions. Iqbal *et al*.^16^ reported similar results from incubation experiments, with a *Q*_10_ of 2.3 in upland soil that was significantly higher than the 1.9 value in paddy field soil. In general, this pattern of *Q*_10_ values could be explained by the fact that SOC in paddy field soil is more labile and the decomposition of labile C components is not as temperature sensitive. The difference in *Q*_10_ values between paddy field and upland soil may also be partly attributed to variation in soil microbial community composition and microbial activity^17^. Chen *et al*.^50^ reported that soil microbial communities and activity levels were significantly influenced by cropland land type, showing that the relative amount of bacterial phospholipid-derived fatty acids (PLFAs), fungal PLFAs, and total PLFAs were greater in paddy fields than in uplands, while the bacterial/fungal PLFA ratio was greater in uplands than in paddy fields.

Under the same incubation conditions and model fitting, even in this study, *Q*_10_ values in the SL were found to be significantly higher than in the TL. These results seem to be consistent with the current prevailing opinion that the decomposition of resistant C is more sensitive to temperature changes^47, 51, 52^. Because significantly higher POXC was found in the TL than in the SL, however, significant negative relationships were observed between *Q*_10_ values and POXC in our study. Fierer *et al*.^53^ and Karhu *et al*.^52^ found that decreasing C quality (degree of resistance to microbial decomposition) with soil depth was responsible for the increase in *Q*_10_ values in grassland and boreal forest soil profiles. A larger proportion of recalcitrant substances in deeper soil have also been shown to significantly increase *Q*_10_ values with increasing depth in a peat ecosystem^18^. Another possible explanation for higher *Q*_10_ values in the SL than in the TL may be the differences in microbial community composition and metabolic activity in the two soil horizons, but this needs to be experimentally verified.

Our results suggest that the differences in SOC decomposition between paddy fields and uplands and between the TL and SL should be considered in soil C models for predicting future C cycling. Furthermore, the results of this study have implications for agricultural management. On one hand, *Q*_10_ values in upland soils were higher than in paddy field soils, suggesting that the upland soils may lose C more easily than the paddy field soil under future global warming. On the other hand, because of the higher *Q*_10_ values in the SL relative to the TL, we should try to ensure shallow tillage because deep plowing could increase air circulation and increase soil temperature in the SL, leading to soil C loss in the SL in a warmer world.

## Conclusions

SOC decomposition and its temperature sensitivity are two important indicators of the character of soil C processes in the context of global climate change. In arable soils, the TL relative to the SL has a significantly higher POXC content and SOC decomposition rate. However, SOC decomposition in the TL is significantly less temperature sensitive than decomposition in the SL. These differences between TL and SL need to be incorporated into soil C models in order to more reliably predict the response of arable soils to global climate change.

## Materials and methods

### Soil sampling and analysis

Cropland soils were sampled from two areas: HP and MS. HP (26°21′–26°58′N, 100°59′–101°31′E) is located in the northwestern part of Yunnan Province, China. The area has a mean annual temperature (MAT) of 19.8 °C and a mean annual precipitation (MAP) of 870 mm. MS (29°34′–30°21′N, 102°49′–104°49′E) is located in Sichuan Province, China, with a MAT of 17.0 °C and MAP of 1236 mm.

Paddy-upland rotation and upland farming are two typical cropping systems in Yunnan and Sichuan because these cropping systems are the major food production methods used in the southwest China. These systems are sensitive to climate change as are other Chinese agricultural ecosystems^54^. Rice, beets, and wheat are the main crops used for paddy-upland rotation, whereas wheat, corn, and soybean are used for upland farming. Six paddy-upland rotation sites (hereafter referred to as paddy field sites) and six upland sites were selected from both HP and MS. Distances between any two sites were about 5 to 10 km of paddy fields or uplands. All sites had been used for conventional farming for over 15 years.

Soil samples were collected in December 2013. Three sampling points (10 m apart) were selected randomly from each site. After surface litter and aboveground plants were removed, an auger with an inner diameter of 8 cm was used to collect soil samples from the 0–20 cm (TL) and 20–40 cm (SL) layers. Xie *et al*.^34^ showed that the average depths of the surface soil layers in Chinese paddy fields and uplands are 15.2 cm and 19.4 cm, respectively. Thus, we established sampling depths of 0–20 cm and 20–40 cm for the TL and the SL, respectively. After the roots were removed, the individual samples from each soil layer at each site were passed through a 2-mm sieve and individually mixed, yielding six TL and six SL samples for paddy field and upland soils in each area, respectively. The soil samples were transferred to the laboratory and stored at 4 °C for analysis and incubation.

Soil water holding capacity (WHC) was gravimetrically determined. Samples were saturated with water, allowed to drain through Whatman #1 filter paper, placed on a glass funnel for 24 h, and then dried at 105 °C for 48 h to a constant weight. Soil pH was measured in a water extract (water to soil ratio of 2.5:1) by using a glass electrode. Soil total carbon (TC) and total nitrogen (TN) were measured using an NC analyzer (FlashEA 1112 Series; Italy). SOC was determined using a TOC analyzer (Multi N/C 3100; Jena, Germany) after removal of carbonates with 0.1 M HCl. Permanganate oxidizable C (POXC) was measured using the method of Culman *et al*.^55^.

### Soil incubation and measurement of respiration

In laboratory incubation, soils have often been incubated at three to five constant temperatures for several months or years (e.g., Conant *et al*.^45^ and Karhu *et al*.^52^). Incubating soils at constant temperatures for a relatively long term in this manner may have biased the estimated *Q*_10_ values of soil respiration, because the substrate, SOC composition, and other soil properties very likely changed with time^42^. We therefore used a short term method of incubating soils (often several days) with changing temperatures^56^. Four replicates of each soil sample, equivalent to a dry weight of about 50 g each, were placed in 250 mL incubation jars. Soil samples were adjusted to 60% WHC and pre-incubated at 20 °C for 72 h to stabilize soil respiration^57^. The incubation jars were then placed in a cryogenic thermostatic bath (DC0530; Bilang Instrument Corp. Ltd., Shanghai). The incubation temperature was initially set to 20 °C, with a step length of 4 °C, increased to 28 °C, and then decreased to 8 °C and back to 20 °C. A similar method using vaying temperature has been reported by Fang *et al*.^56^ and Chen *et al*.^57^. Water loss during the incubation period was periodically checked gravimetrically and adjusted accordingly.

Soil incubation and respiration measurement followed Chen *et al*.^57^. Fresh air via a gas distribution system was continuously passed through the headspace of each incubation jar at a rate of 0.75 L min^−1^. The jars were allowed to remain at each incubation temperature for 2.5 h in order to stabilize soil respiration. Soil respiration was measured by closing the incubation jars and immediately removing a 5-mL gas sample from the headspace of the jar. The same volume of CO_2_-free air was injected into the jar to balance the air pressure. After some time, a second gas sample of 5 ml was obtained, and the incubation jar was opened to allow fresh air circulation. The CO_2_ concentration in the gas samples was measured using a gas chromatograph (Agilent 6890; Agilent Corp., USA) equipped with a flame ionization detector. Soil respiration rate was calculated as the difference in headspace volume, time interval, and CO_2_ concentration between the first and second gas samples^57^, according to the following equation:

1 $${R}_{s}=\,\frac{M}{22.4}\frac{P}{{P}_{0}}\frac{{T}_{0}}{T}\frac{{\rm{\Delta }}C}{{\rm{\Delta }}t}\frac{V}{m}$$

where *R*_s_ is the soil respiration rate (μg CO_2_-C g^−1^ soil h^−1^), *M* is the molar mass of CO_2_-C (g mol^−1^), 22.4 is the molar mass of the gas under standard conditions (273 K, 1013 hPa) (1 mol^−1^), *T*_0_ and *P*_0_ are the temperature (K) and pressure (hPa) of the air under standard conditions, respectively, *T* and *P* are the air temperature (K) and pressure (hPa) at the time of gas sampling, respectively, ∆C/∆t is the change in CO_2_ concentration (ppm) in the jar by time (h), *V* is the headspace volume of jar (l), and m is the dry weight of incubated soil (g).

### Data analysis

The variation in soil respiration rate in response to temperature change was described by an exponential model^57^:

2 $${R}_{s}=a{e}^{bT}$$

where *R*_s_ is the soil respiration rate in *μg* CO_2_-C g^−1^ dry soil h^−1^; *T* is temperature (°C); and *a* and *b* are fitting parameters. Parameter *a* can be referred to as respiration rate at 0 °C, and *b* defines the temperature dependence of soil respiration.

The *Q*_10_ value (the factor of respiration rate increase related to a temperature increase of 10 °C) of soil respiration was then calculated as follows:

3 $${Q}_{10}={e}^{10b}.$$

The MAT was about 17.0 °C and 19.8 °C in MS and HP, respectively. To compare SOC decomposition across soil samples, we chose the decomposition rate at 20 °C because the MAT in the two sampling areas was approximate 20 °C. SOC decomposition at 20 °C, *R*_20_, was defined as

4 $${R}_{20}=a{e}^{20b}.$$

Data were analyzed using a two-factor analysis of variance method to determine the effects of soil horizon and sampling site on SOC, POXC, *R*_20_, and *Q*_10_ in each cropping system (paddy field and upland). All statistical analyses were performed using SPSS 19.0 (IBM/SPSS Inc., Chicago, IL, USA).

## Electronic supplementary material

Supplementary Information

## References

[1] Eswaran H, Vandenberg E, Reich P (1993). Organic carbon in soils of the world. Soil Sci. Soc. Am. J.

[2] Batjes NH (1996). Total carbon and nitrogen in the soils of the world. Eur. J. Soil Sci..

[3] Davidson EA, Janssens IA (2006). Temperature sensitivity of soil carbon decomposition and feedbacks to climate change. Nature.

[4] Lal R (2003). Global potential of soil carbon sequestration to mitigate the greenhouse effect. Crit. Rev. Plant Sci..

[5] Lorenz K, Lal R (2005). The depth distribution of soil organic carbon in relation to land use and management and the potential of carbon sequestration in subsoil horizons. Adv. Agron..

[6] Baisden WT, Parfitt RL (2007). Bomb ^14^C enrichment indicates decadal C pool in deep soil?. Biogeochemistry.

[7] Lal R (2004). Soil carbon sequestration impacts on global climate change and food security. Science.

[8] Smith, P., Fang, C., Dawson, J. J. C. & Moncrieff, J. B. Impact of global warming on soil organic carbon. *Adv. Agron*. **97**, 1–43 (2008).

[9] Lugato E, Berti A (2008). Potential carbon sequestration in a cultivated soil under different climate change scenarios: A modelling approach for evaluating promising management practices in north-east Italy. Agric., Ecosyst. Environ..

[10] Lohila A, Aurela M, Regina K, Laurila T (2003). Soil and total ecosystem respiration in agricultural fields: effect of soil and crop type. Plant Soil.

[11] Fiener P, Dlugoß V, Korres W, Schneider K (2012). Spatial variability of soil respiration in a small agricultural watershed - Are patterns of soil redistribution important?. Catena.

[12] Campos CA (2014). Trends in soil respiration on the eastern slope of the Cofre de Perote Volcano (Mexico): Environmental contributions. Catena.

[13] Bond-Lamberty B, Wang C, Gower ST (2004). A global relationship between the heterotrophic and autotrophic components of soil respiration?. Global Change Biol..

[14] Hanson PJ, Edwards NT, Garten CT, Andrews JA (2000). Separatingroot and soil microbial contributions to soil respiration: A review of methods and observa. Biogeochemistry.

[15] Schlesinger WH (1977). Carbon balance in terrestrial detritus. Annu. Rev. Ecol. Syst..

[16] Iqbal J, Hu R, Lin S, Ahamadou B, Feng M (2009). Carbon dioxide emissions from Ultisol under different land uses in mid–subtropical China. Geoderma.

[17] Thiessen S, Gleixner G, Wutzler T, Reichstein M (2013). Both priming and temperature sensitivity of soil organic matter decomposition depend on microbial biomass – An incubation study. Soil Biol. Biochem..

[18] Hilasvuori E (2013). Temperature sensitivity of decomposition in a peat profile. Soil Biol. Biochem..

[19] Arevalo CBM, Chang SX, Bhatti JS, Sidders D (2012). Mineralization potential and temperature eensitivity of eoil organic carbon under different land uses in the Parkland region of Alberta, Canada. Soil Sci. Soc. Am. J.

[20] Guntinas ME, Gil-Sotres F, Leiros MC, Trasar-Cepeda C (2013). Sensitivity of soil respiration to moisture and temperature. J. Soil Sci Plant Nut..

[21] Chirinda N, Elsgaard L, Thomsen IK, Heckrath G, Olesen JE (2014). Carbon dynamics in topsoil and subsoil along a cultivated toposequence. Catena.

[22] Hamza MA, Anderson WK (2005). Soil compaction in cropping systems: A review of the nature, causes and possible solutions. Soil Till. Res..

[23] Pagliai M, Vignozzi N, Pellegrini S (2004). Soil structure and the effect of management practices. Soil Till. Res..

[24] Radke JK, Dexter AR, Devine OJ (1985). Tillage effects on soil temperature, soil water, and wheat growth in south Australia. Soil Sci. Soc. Am. J..

[25] Kanal A, Kolli R (1996). Influence of cropping on the content, composition and dynamics of organic residue in the soil of the plough layer. Biol. Fert. Soils..

[26] Chen Y, Tcssier S (1997). Techniques to diagnose plow and disk pans. Can. Agr Eng..

[27] Bateman JC, Chanasyk DS (2001). Effects of deep ripping and organic matter amendments on Ap horizons of soil reconstructed after coal strip-mining. Can. J. Soil Sci..

[28] Ball BC, Campbell DJ, Douglas JT, Henshall JK, O’Sullivan MF (1997). Soil structural quality, compaction and land management. Eur. J. Soil Sci..

[29] Curmi P, Merot P, Roger-Estrade J, Caneill J (1996). Use of environmental isotopes for field study of water infiltration in the ploughed soil layer. Geoderma.

[30] Neve SD, Hofman G (2000). Influence of soil compaction on carbon and nitrogen mineralization of soil organic matter and crop residues. Biol. Fert. Soils..

[31] Omonode R, Vyn T (2006). Vertical distribution of soil organic carbon and nitrogen under warm-season native grasses relative to croplands in west-central Indiana, USA. Agric., Ecosyst. Environ..

[32] Doetterl S, Six J, Van Wesemael B, Van Oost K (2012). Carbon cycling in eroding landscapes: geomorphic controls on soil organic C pool composition and C stabilization. Global Change Biol..

[33] Jobbagy EG, Jackson RB (2000). The vertical distribution of soil organic carbon and its relation to climate and vegetation. Ecol. Appl..

[34] Xie Z (2007). Soil organic carbon stocks in China and changes from 1980s to 2000s. Global Change Biol..

[35] Jenkinson DS, Coleman K (2008). The turnover of organic carbon in subsoils. Part 2. Modelling carbon turnover. Eur. J. Soil Sci..

[36] Guimaraes DV, Gonzaga MIS, Neto JDM (2014). Management of soil organic matter and carbon storage in tropical fruit crops. Rev. Bras. Eng. Agr. Amb.

[37] Babujia LC, Hungria M, Franchini JC, Brookes PC (2010). Microbial biomass and activity at various soil depths in a Brazilian oxisol after two decades of no-tillage and conventional tillage. Soil Biol. Biochem..

[38] Raiesi F, Beheshti A (2014). Soil C turnover, microbial biomass and respiration, and enzymatic activities following rangeland conversion to wheat–alfalfa cropping in a semi-arid climate. Environ, Earth, Sci..

[39] van Leeuwen JP (2017). Effects of land use on soil microbial biomass, activity and community structure at different soil depths in the Danube floodplain. Eur. J. Soil Sci..

[40] Cai ZC, Qin SW (2006). Dynamics of crop yields and soil organic carbon in a long-term fertilization experiment in the Huang-Huai-Hai Plain of China. Geoderma.

[41] Kirschbaum M (2000). Will changes in soil organic carbon act as a positive or negative feedback on global warming. Biogeochemistry.

[42] Reichstein M, Bednorz F, Broll G, Kätterer T (2000). Temperature dependence of carbon mineralisation: conclusions from a long-term incubation of subalpine soil samples. Soil Biol. Biochem..

[43] Reichstein M, Subke J-A, Angeli AC, Tenhunen JD (2005). Does the temperature sensitivity of decomposition of soil organic matter depend upon water content, soil horizon, or incubation time?. Global Change Biol..

[44] Holland EA, Neff JC, Townsend AR, McKeown B (2000). Uncertainties in the temperature sensitivity of decomposition in tropical and subtropical ecosystems: Implications for models. Global. Biogeochem. Cycle..

[45] Conant RT (2008). Experimental warming shows that decomposition temperature sensitivity increases with soil organic matter recalcitrance. Ecology.

[46] Zhu B, Cheng W (2011). Constant and diurnally-varying temperature regimes lead to different temperature sensitivities of soil organic carbon decomposition. Soil Biol. Biochem..

[47] Haddix ML (2011). The role of soil characteristics on temperature sensitivity of soil organic matter. Soil Sci. Soc. Am. J..

[48] Moni C (2015). Temperature response of soil organic matter mineralisation in arctic soil profiles. Soil Biol. Biochem..

[49] Leifeld J, Fuhrer J (2005). The temperature response of CO_2_ production from bulk soils and soil fractions is related to soil organic matter quality. Biogeochemistry.

[50] Chen X-J (2013). Microbial activity and community structure analysis under the different land use patterns in farmland soils: based on the methods PLFA and MicroResp. Environ SCI.

[51] Vanhala P (2007). Old soil carbon is more temperature sensitive than the young in an agricultural field. Soil Biol. Biochem..

[52] Karhu K (2010). Temperature sensitivity of organic matter decomposition in two boreal forest soil profiles. Soil Biol. Biochem..

[53] Fierer N, Allen AS, Schimel JP, Holden PA (2003). Controls on microbial CO_2_ production: a comparison of surface and subsurface soil horizons. Global change Biol..

[54] Leng GY, Tang QH, Rayburg S (2015). Climate change impacts on meteorological, agricultural and hydrological droughts in China. Global Planet Change.

[55] Culman SW (2012). Permanganate Oxidizable Carbon Reflects a Processed Soil Fraction that is Sensitive to Management. Soil Sci. Soc. Am. J.

[56] Fang C, Moncrieff JB (2001). The dependence of soil CO_2_ efflux on temperature. Soil Biol. Biochem..

[57] Chen X (2010). Evaluating the impacts of incubation procedures on estimated *Q*_10_ values of soil respiration. Soil Biol. Biochem..

