# Supplementary material for: The temperature sensitivity of soil organic carbon decomposition is greater in subsoil than in topsoil during laboratory incubation
Source: Sci Rep. 2017 Jul 12;7:5181. doi: 10.1038/s41598-017-05293-1 (PMC5507886; doi:10.1038/s41598-017-05293-1)
Supplement: Supplementary file 1 — Supplementary Information [file 41598_2017_5293_MOESM1_ESM.doc]

The temperature sensitivity of soil organic carbon decomposition is greater in subsoil than in topsoil during laboratory incubation

Dong Yan^#^, Jinquan Li^#^, Junmin Pei, Jun Cui, Ming Nie^*^, Changming Fang^*^

Coastal Ecosystems Research Station of the Yangtze River Estuary, Ministry of Education Key Laboratory for Biodiversity Science and Ecological Engineering, The Institute of Biodiversity Science, Fudan University, Shanghai, 200433, PR China

^#^These authors contributed equally to this work.

^*^Corresponding authors: Drs. Ming Nie and Changming Fang

School of Life Science, Fudan University, 2005 Songhu Road, Shanghai, 200438, China

Phone/Fax: +86-21-51630700

E-mail: [mnie@fudan.edu.cn](mailto:mnie@fudan.edu.cn); [cmfang@fudan.edu.cn](mailto:cmfang@fudan.edu.cn)

**Supplementary Table 1** Soil physicochemical properties of the topsoil layer (TL) in six Huaping (HP) paddy field sites

| Site | pH | WHC (%) | SOC (g kg^-1^) | POXC (g kg^-1^) | TN (g kg^-1^) | C/N |
| --- | --- | --- | --- | --- | --- | --- |
| 1 | 7.6 | 29.0 | 12.73 | 2.73 | 1.04 | 15.9 |
| 2 | 8.3 | 30.2 | 15.44 | 2.51 | 1.26 | 16.2 |
| 3 | 8.0 | 26.3 | 17.57 | 3.15 | 1.42 | 16.2 |
| 4 | 8.0 | 27.5 | 14.47 | 2.29 | 1.18 | 16.0 |
| 5 | 8.2 | 25.4 | 12.36 | 1.97 | 1.06 | 15.3 |
| 6 | 8.4 | 27.4 | 11.82 | 2.24 | 0.87 | 17.9 |

**Supplementary Table 2 Soil physicochemical properties of the subsoil layer (SL) in six Huaping (HP) paddy field sites**

| Site | pH | WHC (%) | SOC (g kg^-1^) | POXC (g kg^-1^) | TN (g kg^-1^) | C/N |
| --- | --- | --- | --- | --- | --- | --- |
| 1 | 8.0 | 32.0 | 10.02 | 1.01 | 0.81 | 14.9 |
| 2 | 8.5 | 28.1 | 13.58 | 0.92 | 1.05 | 16.3 |
| 3 | 8.3 | 29.1 | 14.13 | 0.76 | 1.10 | 15.8 |
| 4 | 8.2 | 26.4 | 12.65 | 1.35 | 1.03 | 14.8 |
| 5 | 8.4 | 30.1 | 13.39 | 1.52 | 0.95 | 17.3 |
| 6 | 8.6 | 25.2 | 10.34 | 1.06 | 0.66 | 18.7 |

**Supplementary Table 3** Soil physicochemical properties of the topsoil layer (TL) in six Huaping (HP) upland sites

| Site | pH | WHC (%) | SOC (g kg^-1^) | POXC (g kg^-1^) | TN (g kg^-1^) | C/N |
| --- | --- | --- | --- | --- | --- | --- |
| 1 | 8.8 | 24.2 | 10.34 | 0.59 | 0.95 | 12.7 |
| 2 | 8.4 | 27.8 | 12.33 | 0.88 | 1.08 | 13.4 |
| 3 | 8.5 | 26.0 | 11.37 | 0.94 | 1.05 | 12.8 |
| 4 | 8.8 | 25.1 | 6.73 | 1.03 | 0.59 | 13.4 |
| 5 | 8.5 | 30.6 | 3.68 | 0.48 | 0.35 | 12.7 |
| 6 | 8.2 | 24.8 | 3.54 | 0.55 | 0.31 | 13.3 |

**Supplementary Table 4** Soil physicochemical properties of the subsoil layer (SL) in six Huaping (HP) upland sites

| Site | pH | WHC (%) | SOC (g kg^-1^) | POXC (g kg^-1^) | TN (g kg^-1^) | C/N |
| --- | --- | --- | --- | --- | --- | --- |
| 1 | 8.8 | 28.9 | 6.07 | 0.23 | 0.53 | 13.3 |
| 2 | 8.5 | 27.2 | 7.40 | 0.38 | 0.62 | 13.7 |
| 3 | 8.6 | 29.6 | 7.22 | 0.31 | 0.65 | 12.7 |
| 4 | 8.9 | 32.2 | 2.38 | 0.21 | 0.19 | 13.9 |
| 5 | 8.5 | 26.0 | 2.41 | 0.24 | 0.19 | 14.9 |
| 6 | 8.5 | 30.3 | 1.79 | 0.15 | 0.13 | 15.2 |

**Supplementary Table 5** Soil physicochemical properties of the topsoil layer (TL) in six Meishan (MS) paddy field sites

| Site | pH | WHC (%) | SOC (g kg^-1^) | POXC (g kg^-1^) | TN (g kg^-1^) | C/N |
| --- | --- | --- | --- | --- | --- | --- |
| 1 | 5.7 | 33.4 | 9.59 | 3.41 | 0.80 | 12.7 |
| 2 | 6.0 | 36.2 | 12.93 | 4.21 | 1.11 | 12.4 |
| 3 | 5.8 | 27.7 | 11.17 | 4.32 | 0.94 | 12.2 |
| 4 | 6.2 | 29.1 | 17.94 | 3.82 | 1.39 | 13.1 |
| 5 | 5.8 | 28.0 | 14.26 | 3.03 | 1.23 | 11.8 |
| 6 | 6.1 | 34.2 | 19.65 | 4.08 | 1.69 | 11.8 |

**Supplementary Table 6** Soil physicochemical properties of the subsoil layer (SL) in six Meishan (MS) paddy field sites

| Site | pH | WHC (%) | SOC (g kg^-1^) | POXC (g kg^-1^) | TN (g kg^-1^) | C/N |
| --- | --- | --- | --- | --- | --- | --- |
| 1 | 5.9 | 29.6 | 8.10 | 1.85 | 0.73 | 11.3 |
| 2 | 6.2 | 37.6 | 10.62 | 1.57 | 0.93 | 11.7 |
| 3 | 6.0 | 35.1 | 9.30 | 1.24 | 0.82 | 11.4 |
| 4 | 6.3 | 30.0 | 14.93 | 0.87 | 1.28 | 11.9 |
| 5 | 6.0 | 28.7 | 11.86 | 1.35 | 1.00 | 12.1 |
| 6 | 6.3 | 33.8 | 16.36 | 1.43 | 1.39 | 13.0 |

**Supplementary Table 7** Soil physicochemical properties of the topsoil layer (TL) in six Meishan (MS) upland sites

| Site | pH | WHC (%) | SOC (g kg^-1^) | POXC (g kg^-1^) | TN (g kg^-1^) | C/N |
| --- | --- | --- | --- | --- | --- | --- |
| 1 | 6.3 | 32.1 | 25.86 | 2.98 | 2.17 | 12.9 |
| 2 | 5.9 | 39.2 | 23.53 | 3.18 | 1.93 | 13.1 |
| 3 | 6.3 | 37.1 | 25.04 | 1.99 | 2.04 | 12.7 |
| 4 | 6.8 | 30.1 | 14.75 | 2.15 | 1.08 | 14.4 |
| 5 | 6.1 | 31.0 | 8.70 | 2.76 | 0.65 | 13.8 |
| 6 | 6.4 | 36.7 | 10.14 | 0.86 | 0.76 | 13.8 |

**Supplementary Table 8** Soil physicochemical properties of the subsoil layer (SL) in six Meishan (MS) upland sites

| Site | pH | WHC (%) | SOC (g kg^-1^) | POXC (g kg^-1^) | TN (g kg^-1^) | C/N |
| --- | --- | --- | --- | --- | --- | --- |
| 1 | 6.4 | 27.8 | 20.83 | 1.26 | 1.75 | 12.8 |
| 2 | 6.1 | 36.5 | 23.24 | 1.47 | 1.94 | 12.7 |
| 3 | 6.4 | 34.2 | 22.01 | 1.37 | 1.77 | 12.9 |
| 4 | 6.9 | 37.0 | 11.89 | 1.35 | 0.94 | 13.1 |
| 5 | 6.2 | 29.0 | 5.53 | 0.73 | 0.47 | 12.0 |
| 6 | 6.5 | 36.7 | 8.15 | 0.61 | 0.70 | 12.0 |
